# Editorial: Methods in primary immunodeficiencies: 2022

**DOI:** 10.3389/fimmu.2025.1768176

**Published:** 2026-01-07

**Authors:** Emily S. J. Edwards

**Affiliations:** Department of Immunology, School of Translational Medicine, Monash University, Melbourne, VIC, Australia

**Keywords:** artificial intelligence, inborn errors of immunity, methods, primary immunodeficiencies (PID), treatments

Primary immunodeficiencies (PID), or Inborn Errors of Immunity (IEI), encompass a rapidly expanding group of genetic disorders with diverse clinical manifestations and profound diagnostic challenges. As the field continues to evolve, methodological innovation has become one of the most powerful drivers of discovery to bridge gaps between clinical observation, mechanistic understanding, and translational application. This Research Topic brings together contributions that collectively demonstrate how new methodological approaches are reshaping diagnosis, functional evaluation, and therapeutic monitoring across the PID landscape.

## Emerging diagnostic and treatment frameworks

A number of articles in this Research Topic emphasise the ongoing transformation of PID diagnostics. High-throughput genetic technologies continue to expand in scope and accessibility, and several studies demonstrate how targeted sequencing, whole-exome approaches, and functional genomics are increasingly being integrated into routine clinical workflows. These contributions highlight not only improvements in diagnostic yield but also underscore the importance of stringent variant interpretation, functional validation, and multidisciplinary review, all of which are critical components for ensuring that genetic findings translate into meaningful clinical care.

Complementing genomic advances, this Research Topic also features innovative immunophenotyping techniques. Refined flow cytometry panels, next-generation sequencing of immune repertoires, and sophisticated computational classification strategies enable deeper resolution of immune cell subsets and signalling states. Such tools are indispensable for capturing the heterogeneity of PID and for distinguishing between overlapping phenotypes that traditionally posed diagnostic uncertainty.

## Novel functional immunology methods

Several articles highlight the growing importance of assays that assess immune function beyond static measurements. The article by del Pino Molina et al., examine the technical challenges of intracellular flow cytometry based signalling assays, and their application in the diagnosis of activated PI3K-delta syndrome (APDS). These methodological advances allow researchers and clinicians to define functional defects more precisely, identify immune dysregulation states earlier, and generate mechanistic insights that were not achievable using older techniques. In addition, use of these assays can stratify patients for treatment with targeted therapeutics.

## Data integration and computational approaches

As datasets grow in size and complexity, computational tools have become indispensable. A reviews by Henderson et al. and Soler-Palacin et al., explore machine learning applications, multidimensional data integration, and novel algorithms for clustering immune phenotypes. These approaches enable the field to move beyond single-parameter analyses toward systems-level interpretation of clinical data, immune function and dysfunction towards more rapid diagnosis.

Furthermore, the multicentre study by Avedova et al., demonstrates the importance of simultaneously examining clinical and immunological features of patient responses to subcutaneous immunoglobulin G (SCIg), with quality of life assessments providing key information that patients on SCIg not only achieved stable trough levels of IgG but also exhibited higher physical and mental health score. Thereby, providing crucial support for monitoring these outcomes for patients receiving this life saving treatment.

## Conclusion

The methods highlighted in this Research Topic illustrate the transformative progress occurring in the PID field. Collectively, these articles demonstrate how methodological innovation holds promise for improved diagnostic precision, deepened mechanistic understanding, and support towards the development of personalised treatment approaches. As technologies continue to advance and integrate, they will play an increasingly central role in reducing diagnostic delays, improving therapeutic strategies, and ultimately enhancing quality of life for individuals living with PID.

We thank the authors whose work contributes to this Research Topic, and we anticipate that continued methodological innovation will further accelerate progress in understanding and treating primary immunodeficiencies.

